# Selamectin Is the Avermectin with the Best Potential for Buruli Ulcer Treatment

**DOI:** 10.1371/journal.pntd.0003996

**Published:** 2015-08-13

**Authors:** Nicole Scherr, Gerd Pluschke, Charles J. Thompson, Santiago Ramón-García

**Affiliations:** 1 Department of Medical Parasitology and Infection Biology, Swiss Tropical and Public Health Institute, Basel, Switzerland; 2 University of Basel, Basel, Switzerland; 3 Department of Microbiology and Immunology, Centre for Tuberculosis Research, Life Sciences Centre, University of British Columbia, Vancouver, British Columbia, Canada; Fondation Raoul Follereau, FRANCE

## Abstract

A comprehensive analysis was done to evaluate the potential use of anti-parasitic macrocyclic lactones (including avermectins and milbemycins) for Buruli ulcer (BU) therapy. A panel containing nearly all macrocyclic lactones used in human or in veterinary medicine was analyzed for activity *in vitro* against clinical isolates of *Mycobacterium ulcerans*. Milbemycin oxime and selamectin were the most active drugs against *M*. *ulcerans* with MIC values from 2 to 8 μg/mL and 2 to 4 μg/mL, respectively. In contrast, ivermectin and moxidectin, which are both in clinical use, showed no significant activity (MIC> 32 μg/mL). Time-kill kinetic assays showed bactericidal activity of selamectin and *in vitro* pharmacodynamic studies demonstrated exposure-dependent activity. These data together with analyses of published pharmacokinetic information strongly suggest that selamectin is the most promising macrocyclic lactone for BU treatment.

## Author Summary

Buruli ulcer (BU) is a chronic debilitating mycobacterial disease of the skin and soft tissue caused by *Mycobacterium ulcerans*. It is mainly found in tropical regions and often linked to poverty. BU can be cured in most cases with the standard treatment, a combination of rifampicin and the injectable antibiotic streptomycin. However, new optimized treatment regimens are needed, especially to prepare for an eventual development of resistance to rifampicin, the most efficacious drug for BU therapy. Since traditional antibacterial drug discovery is not a practical option for BU, using approved drugs for alternative clinical indications would be a more economical and faster way to implement new anti-BU therapies. We reported previously that anti-parasitic avermectins are active against *Mycobacterium tuberculosis*. Here we show that some are also active *in vitro* against other mycobacterial species, including *M*. *marinum* and *M*. *ulcerans*. In this study, we undertook a comprehensive approach to evaluate additional macrocyclic lactones including compounds used in veterinary medicine. Based on our *in vitro* measurements of their activities and a literature review of their pharmacokinetic properties, we present strong arguments that selamectin is the avermectin with the highest potential for being repurposed for BU treatment.

## Introduction

Buruli ulcer (BU), caused by *Mycobacterium ulcerans*, is a chronic debilitating disease of the skin and soft tissue. Although mortality is low, permanent disfigurement and disability is high [1]. BU is mainly found in Africa, South America and the Western Pacific regions and is often linked to poverty. If detected early, BU can be cured in most cases with the standard treatment, a combination of rifampicin and the injectable antibiotic streptomycin [2], without further adjunct surgical treatment required. However, new treatment regimens are needed to reduce the long median time to healing, treatment-related side effects, and the requirement for on-site health care workers to administer injections [3]. Furthermore, an alternative drug treatment regimen would be required in the event that rifampicin resistant *M*. *ulcerans* strains would emerge in the clinic [4].

Traditionally, the discovery of new antimicrobial drugs has focused on designing and screening for new compounds having novel targets, an approach that is costly in time and capital (up to ~$800M and 15–20 years) [5]. This is not a viable option for BU, since most large pharmaceutical and biotech companies are primarily interested in blockbuster, broad spectrum antibacterial drugs [6] rather than treatments for neglected tropical diseases. A faster and cheaper alternative to finding new BU treatments is drug repositioning, i.e. using approved drugs for alternative clinical indications [7]. These drugs with known pharmacokinetic and safety profiles could be more rapidly evaluated in clinical trials [8]. Such an approach would also allow for an easier drug introduction, since manufacturing and distribution infrastructures are already available.

In the course of screening clinically approved drugs to find new drug combinations for tuberculosis (TB) therapy, we discovered anti-mycobacterial activities of the avermectins, a class of macrocyclic lactones [9]. Following up these findings, the *in vitro* activities of two clinically approved macrocyclic lactones (ivermectin and moxidectin) against *M*. *ulcerans* were recently reported [10]. The avermectins are a family of macrocyclic lactone derivatives with potent anthelmintic properties, produced by the soil actinomycete *Streptomyces avermitilis*. Since avermectins are inactive against all other bacterial species tested [9], oral administration would not affect healthful intestinal microbiome balances.

We undertook a comprehensive approach to evaluate additional macrocyclic lactones used in veterinary medicine. Based on our *in vitro* measurements of their activities and a literature review of their pharmacokinetic (PK) properties, we provide strong indications that selamectin (used in veterinary medicine), and not ivermectin (used in human medicine), is the avermectin with the highest potential for clinical efficacy to treat BU.

## Materials and Methods

### Bacterial strains, general growth conditions and reagents

*M*. *marinum* isolates (1704 and 1705; kindly provided by Dr. Julian Davies, University of British Columbia) were routinely propagated at 30°C in Middlebrook 7H9 broth (Difco) supplemented with 10% Middlebrook albumin-dextrose-catalase (ADC)(Difco), 0.2% glycerol and 0.05% (vol/vol) Tyloxapol or on Middlebrook 7H10 agar plates (Difco) supplemented with 10% (vol/vol) oleic acid-albumin-dextrose-catalase (OADC)(Difco). *M*. *ulcerans* strains S1012, S1013 and S1047 (isolated in 2010 and 2011 from Cameroonian BU patients) were routinely grown in BacT/Alert culture bottles using enrichment medium (bioMérieux) or on Middlebrook 7H10 agar plates (Difco) supplemented with 10% (vol/vol) OADC. Macrocyclic lactones were purchased from the following providers: abamectin and doramectin (Sigma), emamectin and eprinomectin (LKT Labs), ivermectin (Alpha Diagnostic), milbemycin oxime (US Pharmacopeia), moxidectin and selamectin (European Pharmacopoeia).

### Drug susceptibility assays

Minimal Inhibitory Concentrations (MIC) were determined in 7H9 broth supplemented with 0.2% glycerol and 10% ADC (*M*. *marinum*) or 10% OADC (*M*. *ulcerans*) using two-fold serial dilutions of compounds in triplicate in polystyrene 96-well plates. MTT [3- (4,5-dimethylthiazol-2-yl)-2,5-diphenyl tetrazolium bromide] and resazurin were used as the bacterial growth indicators [11] for *M*. *marinum* and *M*. *ulcerans*, respectively. For *M*. *marinum*, cultures were sampled (100 μL) at a cell density of 10^5^ cells/mL and incubated in the presence of the drug for 3 days before addition of 25 μL of MTT (5 mg/mL). After further overnight incubation, 100 μL of 10% Sodium Lauryl Sulfate (SLS) were added to solubilize the formazan precipitate that indicates bacterial growth and the optical density at 580 nm (OD_580_) was then measured. In the case of *M*. *ulcerans*, 100 μL culture samples (OD_600_ = 0.04) were incubated in the presence of the drug at 30°C for 8 days before addition of 20 μL of a resazurin solution (0.125 mg/mL), followed by overnight incubation at 37°C. Compound activity was determine by fluorescence measurements (λ = 540/588 nm). The lowest concentration of drug that inhibited 90% of the MTT or resazurin color conversion (IC_90_) was used to define MIC values.

### *M*. *ulcerans* kill-kinetic assay

96-well polystyrene plates containing 200 μL per well of 7H9 broth supplemented with 10% OADC were inoculated in duplicate with *M*. *ulcerans* S1013 to a final OD_600_ = 0.04 (ca. 10^5^ cells/mL). Cultures were grown at 30°C in the presence of 0.5, 2, 4, 8, 16 and 32 μg/mL of selamectin (0.5, 1, 2, 4, 8 and 16 fold the selamectin MIC value, respectively) for 0, 3, 7, 14 and 21 days. At every time point, 100 μL of undiluted and ten-fold serial dilutions were plated on 7H10 agar. Colony-forming units for all plates were determined after 8 weeks of incubation at 30°C.

## Results

### Comparative *in vitro* assays of macrocyclic lactones reveal that milbemycin oxime and selamectin are the most potent against *M*. *ulcerans*

**Fig 1:**
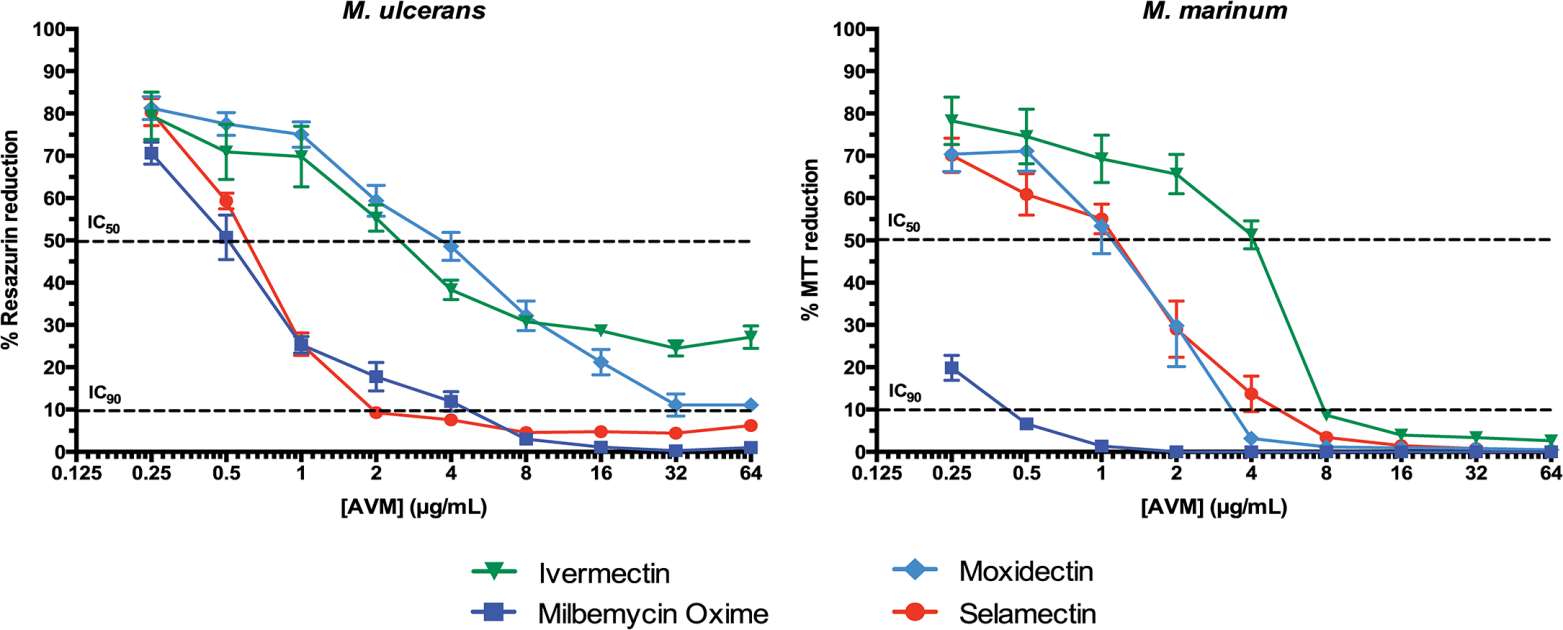
*In vitro* dose-response of the avermectins against *M*. *ulcerans* and *M*. *marinum*. Values are the mean of activities against three *M*. *ulcerans* and two *M*. *marinum* clinical isolates (see Table 1) performed in triplicate. IC_90_ and IC_50_, 90% and 50% reduction of resazurin or MTT color conversion, respectively.

Eight commercially available macrocyclic lactones used in human and veterinary medicine were tested *in vitro* against *M*. *ulcerans* and *M*. *marinum*. Milbemycin oxime and selamectin were the most potent drugs against the *M*. *ulcerans* isolates (MIC in 2–4 μg/mL range). Emamectin and moxidectin had intermediate potency (MIC = ca. 32 μg/mL). While it was not possible to determine minimal inhibitory concentrations for ivermectin (IC_90_ >64 μg/mL), some inhibitory activity was observed in dose response studies. In contrast, most of the macrocyclic lactones showed activity against *M*. *marinum*, a faster growing phylogenetic progenitor of *M*. *ulcerans*, with milbemycin oxime being the most potent (**Fig 1**and **Table 1**).

**Table 1:** Antimicrobial activities of macrocyclic lactones against *M*. *ulcerans* and *M*. *marinum*.

		MIC (μg/mL)^a^							
Strains^b^		Abamectin	Doramectin	Emamectin	Eprinomectin	Ivermectin	Milbemycin oxime	Moxidectin	Selamectin
*M*. *ulcerans*	S1012	>64	>64	32	>64	>64	8	16	2
	S1013	>64	>64	16–32	>64	>64	1–2	>64	1–2
	S1047	>64	>64	32	32	>64	4	32	4
*M*. *marinum*	1704	8–16	16–32	8	32	8	0.25–0.5	2–4	2–8
* *	1705	8–16	32–64	8	32	8–16	0.25–0.5	2–4	4–8

^a^Abamectin, doramectin, emamectin, eprinomectin, ivermectin and selamectin are avermectins; moxidectin and milbemycin oxime are milbemycins.

^b^The resazurin and the MTT methods were used for *M*. *ulcerans* and *M*. *marinum*, respectively.

### Selamectin kills *M*. *ulcerans*

**Fig 2:**
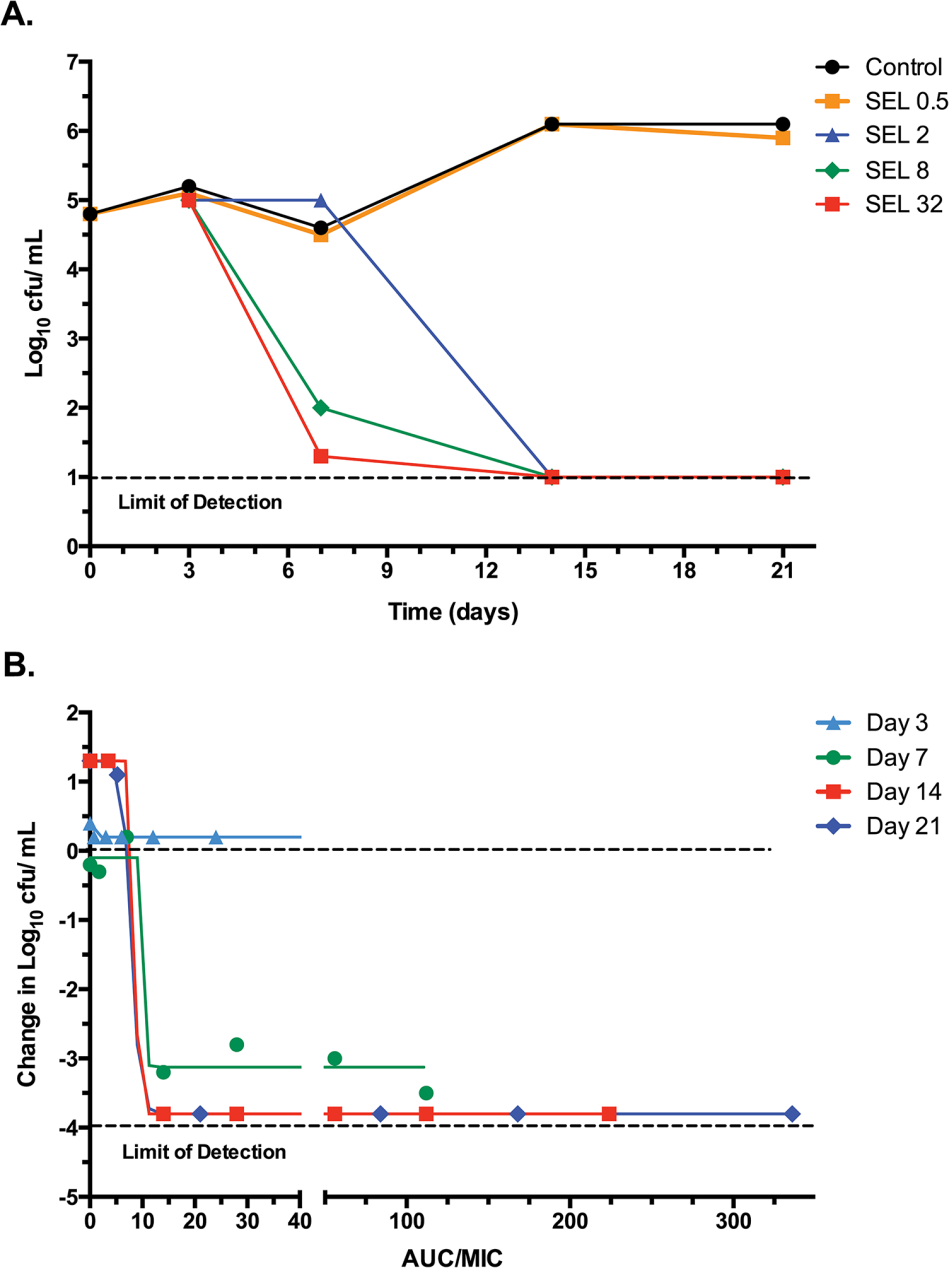
Kill kinetics of selamectin against *M*. *ulcerans* S1013. Dose response kill kinetic assays were plotted based on (**A**) drug concentration and (**B**) incubation time. AUC is the concentration of selamectin multiplied by the time of exposure (AUC = C_SEL_ x T_days_). AUC, area under the curve. SEL, selamectin. Concentrations are in μg/m.

The PK properties of selamectin (described below), together with its high *in vitro* activity against *M*. *ulcerans*, strongly indicated it as the most suitable avermectin for further evaluation as a potential new anti-BU treatment. To further characterize this potential new application, the *in vitro* pharmacodynamic (PD) parameters of selamectin were evaluated using kill-kinetic assays (**Fig 2**). *In vitro* kill-kinetic curves for selamectin were obtained by plotting the number of CFU at every time point for every concentration of the drug (**Fig 2A**). These experiments confirmed the MIC and dose-response data determined by reporters of metabolic activity (resazurin and the MTT; **Table 1**and **Fig 1**) and showed a sharp threshold of bactericidal activity above the MIC (2 μg/mL). We also used an alternative method to visualize kill kinetics: each selamectin concentration was multiplied by the time of exposure (C_SEL_ x T_days_) and then divided by the MIC of selamectin to give the *in vitro* area under the concentration-time curve (AUC/MIC ratio), a standard measure of drug exposure (**Fig 2B**). These analyses showed that just seven days of exposure were needed to observe the bactericidal activity of selamectin. The AUC/ MIC needed to achieve a bactericidal effect (4-log_10_ CFU/ml reduction, 99.99% killing) required AUC/MIC ratios between 10 and 15. These ratios were comparable to those previously observed for *M*. *tuberculosis* [9]. In summary, these studies showed that the activity of selamectin against *M*. *ulcerans* is exposure-dependent; if a certain concentration is achieved, a bactericidal effect is observed by increasing the time of exposure but not by increasing dose concentrations. This information could have important implications when designing pre-clinical and clinical studies.

## Discussion

The family of anthelmintic macrocyclic lactone drugs is one cornerstone of modern parasite control with annual world sales of US $850 million, indicating a well-established production and distribution pipeline. These drugs share a poly-cyclic lactone chemical moiety and can be divided in two sub-families: avermectins and milbemycins [12]. Because members of this family of natural products have complex structures and specificity for parasites, only a few have been commercialized, mostly for veterinary medicine [13]. Ivermectin is used to treat the human parasitic diseases onchocerciasis and lymphatic filariasis [14]. Moxidectin was also recently evaluated for these indications in clinical trials [15]. The potential use of ivermectin for TB treatment is questionable due to its neurotoxicity at high doses and the low exposure levels achieved using clinically approved doses [16]. We analyzed available literature to compare the pharmacological properties of clinically approved drugs (ivermectin, moxidectin) to those with best *in vitro* activities against *M*. *ulcerans* (milbemycin oxime and selamectin)(**Table 2**). By integrating this information with *in vitro* data, we propose selamectin as the anthelmintic macrocyclic lactone with the highest potential for anti-BU therapy.

**Table 2:** Pharmacokinetic meta-analysis summary of selected avermectins and milbemycins.

Drug	Species	Dose (PO unless indicated)	Cmax (ng/mL)	T1/2 (days)	AUC (ng*h/ mL)	Theoretical AUC/MIC^a^	Reference
Ivermectin	Humans	12 mg (165 μg/kg)	47	nd	nd	nd	[17]
		30 mg (fast) (347–541 μg/kg)	85	19 h	2,819	0.04	[18,19]
		30 mg (fed)	260	15 h	4,564	0.07	
		90 mg (1031–1466 μg/kg)	158	19 h	2,910	0.05	
		120 mg (1404–2000 μg/kg)	247	19 h	4,547	0.07	
	Dogs	250 μg/kg	132	80 h	5,600	0.09	[19]
	Horses	200 μg/kg	44	20 h	3,184	0.05	[20]
	Mice (plasma)	0.2 mg/kg	20	9.3 h	573	0.01	[21]
	Mice (lung)	0.2 mg/kg	20	nd	nd	nd	
	Mice	0.2 mg/kg	89.1	nd	711.7	0.02	[22]
Moxidectin	Humans	3 mg (fast)	22.4	33.8	1,442	0.04	[23]
		9 mg (fast)	57.9	34.6	3,024	0.10	
		18 mg (fast)	141	22	5,856	0.18	
		36 mg (fast)	289	20.2	10,824	0.34	
		36 mg (fed)	296	25.7	14,976	0.46	
	Dogs	250 μg/kg	234	621 h	11,800	0.36	ProHeart6 (Product profile)
	Mice	0.2 mg/kg	47.4	nd	643.7	0.02	[22]
	Cattle (plasma)	0.2 mg/kg (s.c)	35.6	8.9	159 (ng.d/g)	0.004	[24]
	Cattle (lung)	0.2 mg/kg (s.c)	63.7	9.1	298 (ng.d/g)	0.008	
Milbemycin oxime		0.25 mg/kg	79.33	11.09	nd		[25]
		0.92 mg/kg (once monthly for 3 mo)	353	67.9	6,754.9	3.4	[26]
		1.19 mg/kg	152	50.2	3,620	1.8	
		1.10 mg/kg	199	58.2	5,165	2.6	
Selamectin	Dogs (male)	6 mg/kg (topical)	12.72	12.14	4,609	2.3	Stronghold (Product profile)
	Dogs (female)	6 mg/kg (topical)	22.65	10.73	8,903	4.45	
	Mice (plasma)	12 mg/kg	3,714	5.5	62,285.7	31.14	[21]
	Mice (lung)	12 mg/kg	7,500	nd	nd	nd	
	Rats	10 mg/kg	>1,000	10.3 h	nd	nd	Stronghold (Product profile)
	Dogs	24 mg/kg	7,630	45.7 h	227,901	113.95	[27]
		24 mg/kg (topical)	86.5	266	15229	7.61	
		95 mg/kg	nd	nd	nd	nd	[28]
	Cats	24 mg/kg	11,929	97.7	1,109,933	554.97	[27]
		24 mg/kg (topical)	5,513	198	743,349	371.67	

^a^Theoretical AUC/MIC was calculated based on our *in vitro* MIC values of the avermectins against *M*. *ulcerans*. MIC values (in ng/mL) used for calculations were: ivermectin, 64,000; moxidectin, 32,000; milbemycin oxime, 2,000, and selamectin: 2,000.

In invertebrate nematodes, avermectins specifically bind to glutamate-gated chloride channels present in nerve and muscle cells, causing paralysis and reduced ability to reproduce. In general, macrocyclic lactones have a high margin of safety in mammals because P-glycoproteins (P-gp) or other types of efflux pumps, highly expressed at the blood–brain barrier, efficiently restrict their penetration into the central nervous system. In fact, dogs lacking the MDR1 efflux pump, such as collies, have much less tolerance for treatment with an array of avermectin compounds [29]. In contrast, milbemycin oxime, selamectin, and moxidectin can be safely administered at therapeutic doses to dogs having a homozygous MDR1 mutation without any signs of toxicosis [12,28].

PK and toxicological profiles of the clinically used macrocyclic lactones (ivermectin and moxidectin) have been studied extensively. Using standard dosages for onchocerciasis treatment in humans, ivermectin is extremely well tolerated, effective, orally active, and associated with long-term safety at the current clinical dose (single dose of 12 mg) [17]. Clinical studies have shown that it is safe in humans at doses up to 10-fold higher; however, further increased dosage provokes severe neurotoxicity [18]. To catalyze application of ivermectin’s therapeutic potential in needy areas throughout the world, Merck & Co. has donated it for over 20 years to treat patients with river blindness, human onchocerciasis, and lymphatic filariasis [14]. In the case of moxidectin, single doses of up to 36 mg were safe in humans, but not doses of 54 mg [23].

The extensive use of macrocyclic lactones in veterinary medicine has generated valuable pharmacological data that could guide selection of these drugs and facilitate their use in humans. Milbemycin oxime is a broad-spectrum intestinal anti-parasitic drug used to treat roundworm, hookworm and tapeworms in cats and dogs; it is also reported to be safer than ivermectin [12]. Administered routinely at a dose of 0.25 mg/kg, it showed no signs of toxicity [25]. Although LD_50_ values after oral administration in dogs are higher than 200 mg/kg, a single dose of 3.8 mg/kg was reported to cause reversible neurological signs (trembling, ataxia) in dogs [30]. In contrast, selamectin has fewer neurological side effects, and can be administered topically, subcutaneously, or orally to treat a variety of ecto- and endo-parasitic infections in cats and dogs. It is the drug of choice in avermectin-sensitive collies since it has no adverse effects [REVOLUTION—fact sheet]. A toxicity study in female CD1 mice found that selamectin was well tolerated at up to 300 mg/kg body weight (bw), while similar doses of milbemycin oxime were toxic [31]. In the case of milbemycin, doses up to 24 mg/kg bw were safe in cats and dogs [27] and one study reported that doses up to 94 mg/kg bw were safe in dogs [28]. In addition, a 3-month repeated dose toxicity study in dogs found an oral dose of 40 mg/kg/day to be safe [28]. Extrapolated to humans, this corresponds to a dose of 2,800 mg/day (for a 70 kg adult). Confirming this extrapolation, the LD_50_ in rats and mice could not be demonstrated and it was higher than 1,600 mg/kg bw [Stronghold (selamectin)—Product profile].

Based on established clinical experience in humans at low dosages, Omansen *et al*. [10] chose to study the anti-mycobacterial activities of ivermectin and moxidectin. They reported MIC values between 4 and 8 μg/mL against *M*. *ulcerans* and inactivity (MIC ≥32 μg/mL) against *M*. *marinum*. We confirmed the activities of macrocyclic lactones, but found different specificities against bacterial isolates representing these two species (**Table 1**and **Fig 1**). In contrast to analyses reported by Omansen *et al*. [10], we detected little or no activity of ivermectin and moxidectin against *M*. *ulcerans* isolates but they were active against *M*. *marinum* strains. Such discrepancies could reflect variations in methodology. While Omansen *et al*. used Mycobacteria Growth Indicator Tubes (MGIT) and bioluminescence assays for their inhibitions assays [10], we performed metabolic-based activity assays in liquid cultures grown in 96 well plates. Subtle differences in methodology are known to play a critical role in quantification of the anti-mycobacterial activity of ivermectin [16].

Our *in vitro* results can be integrated with available PK data to predict which drug would be more suitable for anti-BU therapy. While no human data are available for milbemycin oxime and selamectin, extensive pharmacological data from animal studies provide valuable information to accelerate clinical testing. Standard oral doses (in μg/kg bw range) of ivermectin, moxidectin and milbemycin oxime used to treat helminths in humans only achieve low concentrations in the plasma (ng/mL range). Area Under the Curve (AUC) values for moxidectin and milbemycin oxime are higher than those of ivermectin, mainly due to their extended residence times (higher half-life). However, the much higher doses needed to achieve concentrations sufficient to kill mycobacteria might not be possible due to toxicity. In contrast, selamectin toxicity is negligible at comparable doses. Standard dose administration of selamectin is in the mg/kg bw range (versus μg/kg bw) and doses as high as 95 mg/kg bw have been administered without any side effects [28]. The ability to deliver such high doses without toxicity is also reflected in the elevated concentrations of selamectin that can be achieved in the plasma. These concentrations in the μg/mL range are several fold higher than MIC values against *M*. *ulcerans* [21,27] which, together with a long half-life (in days), allows for high AUC values. In fact, AUC/MIC values are the most predictable PK/PD parameter for the anti-mycobacterial activity of the avermectins [9]. Similarly, AUC/MIC ratios between 10 and 15 are also needed for bactericidal activity against *M*. *ulcerans* (**Fig 2B**). Thus, when theoretical AUC/MIC values were calculated by integrating data from available PK literature with those from our *in vitro* data, only selamectin was predicted to have therapeutic activity against *M*. *ulcerans* (**Table 2**)(nb, calculations based on the lower *in vitro* MIC measurement reported by Omamsen et al. [10] generated the same conclusion). We would also like to point out that when we made corresponding calculations based on *in vitro* MIC data for *M*. *tuberculosis* [9], selamectin would also be the avermectin of choice for tuberculosis therapy.

A synergistic interaction between rifampicin and ivermectin against *M*. *ulcerans* has also been reported [10]. Rifampicin is the cornerstone drug for BU treatment. Thus, co-administration of rifampicin with any synergistic, orally available drug would be ideal. Rifampicin is an inducer of the P-gp and other transporters. P-gp protects mammals not only by excluding macrocyclic lactones from the central nervous system, but also by limiting the uptake of compounds from the gastrointestinal tract and by promoting their excretion in the liver, kidney, and intestine. While ivermectin is a good P-gp substrate, thus further reducing available levels of this drug, selamectin is a poorer P-gp substrate [12,21] and its plasma levels would be affected to a lesser extent allowing for a potential co-administration with rifampicin.

In summary, drug repositioning is an interesting avenue to provide new treatments for neglected diseases. We have tested the family of commercially available macrocyclic lactones against *M*. *ulcerans* and *M*. *marinum* and demonstrated that milbemycin oxime and selamectin are the most active drugs (MIC = 2 μg/mL). Integrating these values with information gathered in a literature review of the pharmacological properties (toxicity and PK/PD profiles) of ivermectin, moxidectin, milbemycin oxime and selamectin, revealed selamectin as the most promising avermectin candidate for anti-BU treatment. Although selamectin is not approved for use in humans, extensive information is available on its pharmacological properties in animals, thus facilitating its progression into clinical trials. These would be warranted if its activity could be validated using *in vivo* models of *M*. *ulcerans* infection. Pre-clinical and clinical development of any drug is a task that one research group cannot achieve alone. Thus, we urge collaboration among the research communities, pharmaceutical companies, and non-governmental organizations to validate the potential of macrocyclic lactones, especially selamectin, as a new anti-BU treatment.
